# Overlapping Community Detection Based on Attribute Augmented Graph

**DOI:** 10.3390/e23060680

**Published:** 2021-05-28

**Authors:** Hanyang Lin, Yongzhao Zhan, Zizheng Zhao, Yuzhong Chen, Chen Dong

**Affiliations:** 1 School of Computer Science and Communications Engineering, Jiangsu University, Zhenjiang 212013, China; linyanhanson@163.com; 2 Jiangsu Start Dima Data Processing Co., Ltd., Kunshan 215217, China; 3 College of Mathematics and Computer Science, Fuzhou University, Fuzhou 350108, China; zhzz7364@gmail.com (Z.Z.); yzchen@fzu.edu.cn (Y.C.); dongchen@fzu.edu.cn (C.D.)

**Keywords:** attributed networks, augmented attribute graph, community detection

## Abstract

There is a wealth of information in real-world social networks. In addition to the topology information, the vertices or edges of a social network often have attributes, with many of the overlapping vertices belonging to several communities simultaneously. It is challenging to fully utilize the additional attribute information to detect overlapping communities. In this paper, we first propose an overlapping community detection algorithm based on an augmented attribute graph. An improved weight adjustment strategy for attributes is embedded in the algorithm to help detect overlapping communities more accurately. Second, we enhance the algorithm to automatically determine the number of communities by a node-density-based fuzzy k-medoids process. Extensive experiments on both synthetic and real-world datasets demonstrate that the proposed algorithms can effectively detect overlapping communities with fewer parameters compared to the baseline methods.

## 1. Introduction

Complex patterns exist in various real-world fields and can be simplified into complex networks. Individuals are represented as nodes, and the connections between them are correspondingly transformed to edges in a graph [1,2,3]. For example, the connections between proteins in organisms and the relationship between cities in a traffic system [4]—these real complex systems can be transformed into complex networks. Community detection is a common task in the field of complex network analysis [5,6].

Many studies have attempted to incorporate attribute and topology information in the community detection methodologies [7] beyond the traditional approaches [8,9]. In addition to the topological structure of nodes connected by edges, the nodes or edges themselves always carry attribute information—that is, they form an attributed network. The attributes can be used as complementary information to overcome the sparsity of topological structure [10,11]. However, these two sources of information may be contradictory to each other in some cases [12]. This makes it challenging to detect communities on attributed networks. In real-world networks, there may exist some vertices that belong to several communities simultaneously. Consequently, overlapping community detection has become a valuable research topic [13]. The proposed OCEA and AOCEA are implemented over attributed networks and can be used to detect overlapping communities. Most of the real-world networks are full of attribute information and overlapping communities are quite common in social networks. Therefore, the proposed algorithms will have a wide range of applications in real life.

The main contributions of this work are as follows: (1) We proposed two algorithms OCEA and AOCEA that can be used to detect overlapping communities on attributed networks, which considers the multiple vertex-community memberships and employs a strategy to adjust attribute weights iteratively according to the memberships. (2) A new method for the automatic estimation of the number of communities is proposed to improve the practicability of OCEA. (3) Experimental results on synthetic and real-world datasets validate the effectiveness of OCEA and AOCEA.

Related work for Big Data Networks. Researches on community detection in large-scale networks are motivated by the situation that the traditional methods cannot handle the increasing size of networks. ADVNDS [14] utilized modularity maximization and designed a heuristic method to solve it. The algorithm proposed in [15] combined parallel processing techniques with binary trees to solve the efficiency problem. The algorithm proposed in [16] extended it later using CPU-GPU modules. CDMEC [17] introduced several functions to construct similarity matrices and integrated a stacked autoencoder and transfer learning to learn the embeddings of large-scale networks.

Related work on Attributed Networks. From the perspective of processing attribute information, existing algorithms can be classified into five categories [18]. First, algorithms based on distance design a distance function to combine attribute topology information. SToC [19] made use of the Jaccard index and Euclidean distance to measure the similarity of topology or attribute information, respectively. SA-cluster [20] obtains the distance by applying a random walk on an augmented attribute graph, and Inc-cluster [21] is a time-saving version of SA-cluster. Algorithms based on representation learning mainly focus on the process of learning the low-dimension vectors of nodes—that is, the embeddings. Community detection can be performed directly through clustering by embeddings. Potential information in the network can then be fully utilized by this method. MGAE [22] proposes a marginal graph convolutional network and obtained deeper representations through multiple autoencoders. DANE [23] employs two sub-processes to learn the attribute and topology representation. The final result is obtained by minimizing a designed negative log-likelihood. Evolutionary-algorithm-based methods measure the similarity of the topology and attribute information, and then transform the problem of community detection into a multi-objective optimization problem. BBO [24] proposes Simatt to represent the similarity of node attributes. Similarly, MOEA-SA [25] proposes SA to measure the attribute information. Nonnegative matrix factorization can also be used to obtain the representation of nodes. SCI [10] proposes a non-negative matrix factorization model with two sets of parameters. SCD [26] introduces an additional community relationship indicator matrix. The elements of the matrix describe the relationship between the corresponding communities. ASCD [27] introduces the concept of a mismatch between attributes and structural information, and then related adaptive parameters were added to detect communities. Finally, probabilistic generative model-based algorithms focus primarily on obtaining a generative model of communities. They directly transform the complex network structure into a probability model determined by several parameters. CESNA [28] models the interaction between a network structure and attributes to detect overlapping communities. The NEMBP [29] model utilizes nesting EM algorithms and confidence propagation to detect the communities based on the correlation between topology and attribute.

Related work on Overlapping Community Detection. The recent overlapping community detection algorithms can be classified into five categories. First, algorithms based on multi-objective evolutionary approach the global optimal solutions by swarm evolution [30,31,32]. MR-MOEA [31] introduces a mixed representation that consists of all the potential overlapping vertices and all the non-overlapping vertices. They evolve together to detect communities. MOGA-OCD [32] uses measures related to network connectivity to optimize two objectives: maximizing internal connectivity and minimizing external connectivity. Algorithms based on similarity partition vertices into communities according to their mutual similarity. OCDDP [33] proposes a method based on density peaks. LED [34] transforms the similarity into weights of the networks. Algorithms based on local expansion first select initial vertices and then expand them to obtain communities. [35] optimizes the conductance community score to determine good seeds and then greedily expands them. [36] aims to find the structural centers of communities. Algorithms based on random walk utilize the path of random walk to define the connectivity among individuals. MCLC [37] employs a random walk on the edges and obtains “link communities” that are transformed into overlapping “node communities.” Finally, algorithms based on representation learning are similar to those based on attributed networks. Through underlying community membership, CDE [38] formulates community detection as a non-negative matrix factorization model based on the encoded community structures and attributes.

It is still challenging to detect overlapping communities on attribute networks. As introduced above in related work, only CESNA [28] and CDE [38] can solve the problem. To solve the problem, an overlapping community detection algorithm based on attribute augmented graph, OCEA, is proposed. We employ fuzzy k-medoids [39] on attribute augmented graph first proposed by [20] to obtain the communities. Furthermore, the number of communities *k* can be evaluated using the density of vertices. Through the evaluation, the automatic OCEA can detect overlapping communities without parameter *k* and obtain comparable or even better results comparing to the baseline methods.

The remainder of this paper is organized as follows. Section 2 introduces some preliminary information about the clustering problem and related definitions. Section 3 discusses the details of OCEA and its automatic variant. Section 4 presents empirical studies of the proposed algorithms. Finally, the conclusion and potential future work are reported in Section 5.

## 2. Preliminaries

### 2.1. Problem Definition

**Figure 1:**
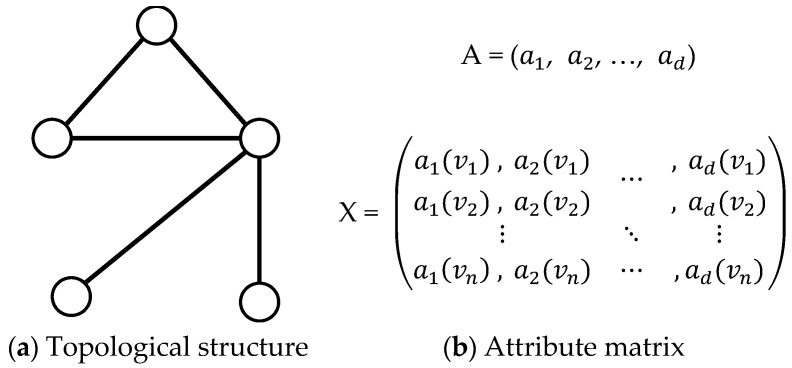
Illustration of an attributed network. For attribute matrix $X\in R^{n\times d}$, $X_{ii}=1$ when vertex *i* has the attribute *m*, otherwise $X_{im}=0$.

Attributed network can be denoted as *G* = (*V*, *E*, *A*, *X*), where *V* = {*v*_1_, *v*_2_, …, *v_n_*} is a set with *n* vertices, *E* = {(*v_i_*, *v_j_*)|*v_i_*, *v_i_*∈*V*} is a set of edges, *A* = {*a*_1_, *a_2_, …, a_d_*} denotes the set of attributes, and *X* = (*x*_1_, *x*_2_, …, *x_n_*)*^T^* is an attribute matrix. Each row of *X* denotes the binary attribute vector of vertex *i* with *d* dimensions. If vertex *i* has attribute *j*, *a_j_*(*v_i_*) = 1. Otherwise, *a_j_*(*v_i_*) = 0. Figure 1 shows a traditional formulation of an attributed network. The attribute information of a vertex or an edge is regarded as a *d*-dimensional binary vector associated with the vertex or edge. In this study, we utilize a different formulation called the augmented attribute graph first proposed by [20] to detect overlapping communities on attributed networks.

The overlapping community detection problem on attributed networks can be discussed from two aspects:

- **Overlapping community:** In a traditional community detection problem, the final partitions do not share any vertices with each other. However, some vertices may be assigned to multiple communities in an overlapping community detection problem. In this paper, we utilize the framework of fuzzy k-medoids [39] to calculate the memberships of every vertex to communities and finally obtain the overlapping communities.
- **Attributed network:** For an attributed network, the final partitions should satisfy two properties: (1) Topology similarity. The vertices belonging to the same community have more connections with each other than the vertices outside the community. (2) Attribute homogeneity. The vertices whose attribute vectors are close to each other have a high probability to be assigned to the same community. The community partitions should embody both the topology similarity and attribute homogeneity.

### 2.2. Augmented Attribute Graph

**Figure 2:**
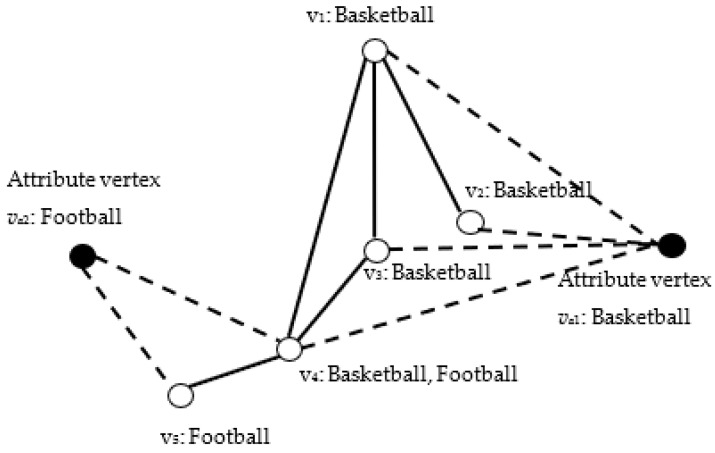
Example of augmented attribute graph.

In contrast to the traditional formulation of an attributed network, the augmented attribute graph directly transforms the attributes into attribute vertices and adds them to the original graph. Figure 2 shows an example of an augmented attribute graph, the set of attributes *A =* {Football, Basketball}, and the set of structure vertices *V* = {*v*_1_, *v*_2_, …, *v*_5_}. The solid points denote the added set of attribute vertices *V_a_ =* {*v_a_*_1_, *v_a_*_2_}, and the hollow points represent the original topology structure of the network. The solid lines denote the original structure edges. The hollow lines are used to connect the attribute and structure vertices. Vertex *v*_4_, for example, has two attributes: Basketball and Football. Thus, there are two attribute edges connected to the attribute vertices *v_a_*_1_: Basketball and *v_a_*_2_: Football. Consequently, the attribute vectors can be transformed into attribute vertices and edges on the augmented attribute graph.

Due to the addition of attribute vertices, the structure similarity and attribute homogeneity between vertices can be uniformly represented by the probability of random walks on the augmented attribute graph. Based on this, there are four cases to calculate the transition probability of random walk. The corresponding equations are expressed as follows:

1. From structure vertex *v_i_* to structure vertex *v_j_*:
  (1) $$p_{v_{i},v_{j}}=\{\begin{array}{cc} \frac{\omega_{0}}{N\left(v_{i}\right)\omega_{0}+\omega_{1}+\omega_{2}+\ldots +\omega_{d}}, & if\left(v_{i},v_{j}\right)\in E\\ 0, & \;\mathrm{otherwise}\; \end{array}$$
2. From attribute vertices *v_ip_* and *v_jq_* corresponding to the *p*th attribute of vertex *v_i_* and the *q*th attribute of vertex *v_j_*, respectively:
  (2) $$p_{v_{ip},v_{jq}}=0,\forall v_{ip},v_{jq}\in V_{a}$$
3. From attribute vertex *v_ip_* to structure vertex *v_j_*:
  (3) $$p_{v_{ip},v_{j}}=\{\begin{array}{c} \frac{1}{N\left(v_{i}\right)},if\left(v_{ip},v_{j}\right)\in E_{a}\\ 0,\;\;\mathrm{otherwise}\; \end{array}$$
4. From structure vertex *v_i_* to attribute vertex *v_jq_*:
  (4) $$p_{v_{i},v_{jq}}=\{\begin{array}{c} \frac{\omega_{j}}{N\left(v_{i}\right)\omega_{0}+\omega_{1}+\omega_{2}+\ldots +\omega_{d}},\;\mathrm{if}\;\left(v_{i},v_{jq}\right)\in E_{a}\\ \;\;\;\;0,\;\mathrm{otherwise}\; \end{array}$$
  where *d* is the dimension of attribute vectors. *ω* denotes the weights of the topology and attribute information, $\omega_{0}$ denotes the weight of topology information, and $\omega_{1}{,\;\omega }_{2}{,\;\ldots ,\;\omega }_{d}\;$ denote the weights of attribute information. *N*(*v_i_*) denotes the number of neighbors of $v_{i}$ on the original network. The indices *p* and *q* in Equations (2)–(4) could be equal or not, and $p,\;q\;\in {\;\{a}_{1}{,\;a}_{2}{,\ldots ,\;a}_{d}\}$.

Based on this, the transition probability of *l* step random walks between each pair of vertices can be obtained by Equation (5).

(5) $$R_{A}=\sum_{\gamma =1}^{l}c{\left(1-c\right)}^{\gamma }P_{A}^{\gamma }$$

where *c* is the stop probability of the random walk, *l* is the step length and *γ* is a power. *P_A_* is the transition probability matrix calculated by the Equations (1)–(4). Equation (5) denotes the process of *l* step random walks on the augmented attribute graph with a stop probability *c*. Here $R_{A}$ is considered the similarity between vertices that reflects both topology and attribute information on the network.

## 3. Algorithms

**Figure 3:**
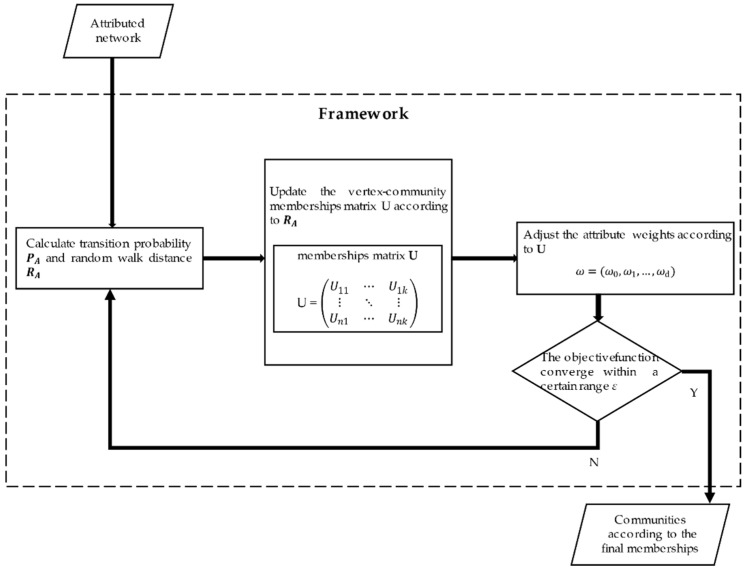
Framework of OCEA.

Here, we introduce the proposed OCEA based on augmented attribute graph and its extended version with an estimation of the number of communities. The framework of OCEA is shown as Figure 3. 

As shown in Figure 3, OCEA is mainly composed of three steps. First, it calculates the transition probability matrix $P_{A}$ and the random walk similarity matrix $R_{A}$. Second, the vertex-community membership matrix *U* is updated according to *R_A_*. Third, the structural weight *ω*_0_ is fixed to 1, and the attribute weights $\omega_{1}{,\;\omega }_{2}{,\;\ldots ,\;\omega }_{d}$ are updated according to matrix *U*. The procedure is repeated until the objective function converges within a certain range *ε*.

### 3.1. Overlapping Community Detection

Based on the framework of the augmented attribute graph, the OCEA utilizes fuzzy k-medoids [39] to detect overlapping communities. *i*th row of the random walk similarity matrix *R_A_* is used as the vector of *v_i_* that is denoted as *R_A_*(*v_i_*). Before the iteration of updating memberships, *k* initial vertices are selected as the centers of each cluster.

Equations (6) and (7) show the process of updating memberships matrix *U* and the vector $X_{c}$ for centers in each cluster:

(6) $$U_{ij}^{\left(t\right)}=\frac{1}{\sum_{c=1}^{k}{\left(\frac{\left\|R_{A}^{\left(t\right)}(v_{i})-X_{c}^{\left(t\right)}(j)\right\|}{\left\|R_{A}^{\left(t\right)}(v_{i})-X_{c}^{\left(t\right)}(k)\right\|}\right)}^{2}}$$

(7) $$X_{c}^{\left(t+1\right)}(j)=\frac{\sum_{i=1}^{N}{\left(U_{ij}^{\left(t\right)}\right)}^{m}\cdot R_{A}^{\left(t\right)}(v_{i})}{\sum_{i=1}^{N}{\left(U_{ij}^{\left(t\right)}\right)}^{m}}$$

where $U_{ij}^{\left(t\right)}$ denotes the memberships of vertex $v_{i}$ to cluster *j*. Further, $X_{c}\left(j\right)$ is not the vector of an actual existing vertex that is regarded as the center of cluster *j*. It denotes the average vector of all vertices in cluster *j*. And $X_{c}\left(j\right)$ is used to update the memberships of all vertices in cluster *j*. Thereafter, the center vertices of each cluster are selected as follows:

(8) $$c_{i}^{\left(t+1\right)}=\arg\min_{v_{j}\in V_{i}}\sum_{vi\in V_{i}}\frac{\left\|R_{A}^{\left(t\right)}(v_{j})-R_{A}^{\left(t\right)}(v_{i})\right\|}{\left|V_{i}\right|}$$

The main objective of the algorithm is to ensure the vertices are close to their corresponding cluster centers. To this end, the objective function is minimized by

(9) $$min\;F=\sum_{i=1}^{N}\sum_{j=1}^{k}U_{ij}^{m}{\left\|R_{A}(v_{i})-R_{A}(c_{j})\right\|}^{2}$$

where *c_j_* denotes an actual existing vertex that is the center of cluster *j* determined by Equation (8). It is different from $X_{c}\left(j\right)$ in Equation (7). The *m* in Equation (7) and Equation (9) is a parameter and it will be set to 2 in experiments. The Euclidean distance is used to measure the similarity between vertices, after which the vertices are assigned to different communities according to their memberships to the communities.

### 3.2. Weights Adjustment

Each iteration of the update can obtain memberships. The weights of each attribute can be adjusted based on the currently detected communities. The equations of attribute weights adjustment are as follows:

(10) $${\mathrm{weight}}_{p}\left(c_{j},v_{i}\right)=\{\begin{array}{l} U_{ij},\;\mathrm{if}\;c_{j},v_{i}\;\mathrm{share}\;\mathrm{the}\;\mathrm{same}\;\mathrm{value}\;\mathrm{on}\;a_{p}\\ 0, \end{array}$$

(11) $$\omega_{i}^{t+1}=\frac{1}{2}\left(\omega_{i}^{t}+\frac{d\sum_{j=1}^{k}\sum_{v\in V_{j}}{\mathrm{weight}}_{i}\left(c_{j},v\right)}{\sum_{p=1}^{d}\sum_{j=1}^{k}\sum_{v\in V_{j}}{\mathrm{weight}}_{p}\left(c_{j},v\right)}\right)$$

Memberships can be observed as the influence of a vertex on a cluster. The high similarity of a certain attribute in a cluster implies that the attribute is an effective feature to detect communities. Subsequently, the weights of the corresponding attributes should be increased, or else they should be decreased.

### 3.3. Estimation on the Number of Communities

In the case of community detection without ground truth, we cannot obtain the number of communities *k* directly. Most algorithms need to input *k* as a hyperparameter or directly set *k* as a fixed value. Based on OCEA, the automatic version of it is proposed using a process for estimating the number of communities *k*. It will be called AOCEA for brevity in the following.

First, the density of vertex is defined by Equation (12):

(12) $$D(v_{i})=\sum_{v_{j}\in V}\left(1-e^{-\frac{R_{A}{\left(v_{i},v_{j}\right)}^{2}}{2}}\right)$$

**Figure 4:**
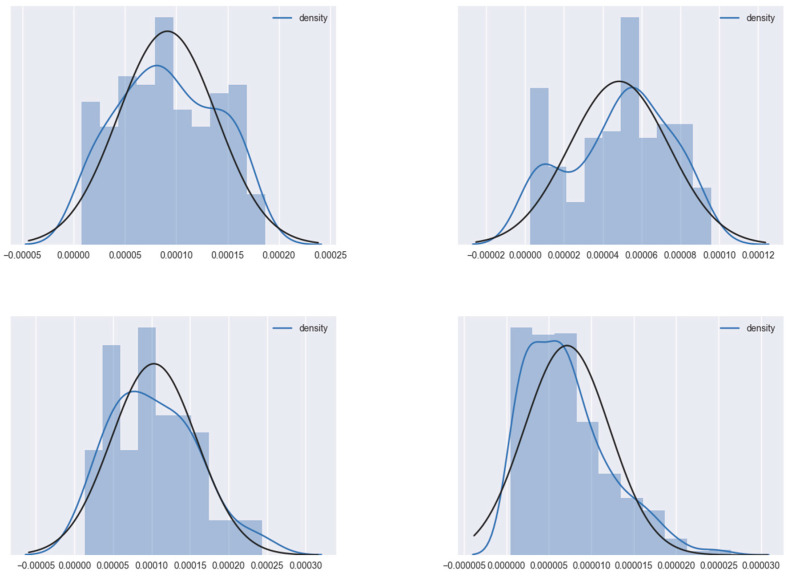
Probability distribution curve of four Facebook egonetworks’ density values.

Taking out four egonetworks randomly from the Facebook egonetwork set introduced in Section 4.1, we draw the histogram of the probability density distribution of each egonetwork’s vertex density value in Figure 4. When calculating the density, the structural weight $\omega_{0}$ is set to 1, and the attribute weights $\omega_{1}{,\;\omega }_{2}{,\;\ldots ,\;\omega }_{d}$ are set to 1/*d*. The stop probability *c* is set to 0.5 to draw the curves.

The x-axis denotes the vertex density value and the y-axis denotes the number of vertices. Each blue curve denotes the probability distribution curve fitted to each histogram. Each black curve denotes the Gaussian distribution calculated according to the mean and standard deviation of each subnetwork’s vertex dense value, as shown in Table 1.

As we can observe in Figure 4, the fitted curves are very close to their corresponding calculated Gaussian distribution curves, which means that each sub-network’s vertex density value is nearly Gaussian distributed. It is assumed that centers should have higher density values than other vertices—higher density values imply a higher probability of being a center. Therefore, the sample mean of the density value is used as the threshold for selecting the candidate initial centers.

**Figure 5:**
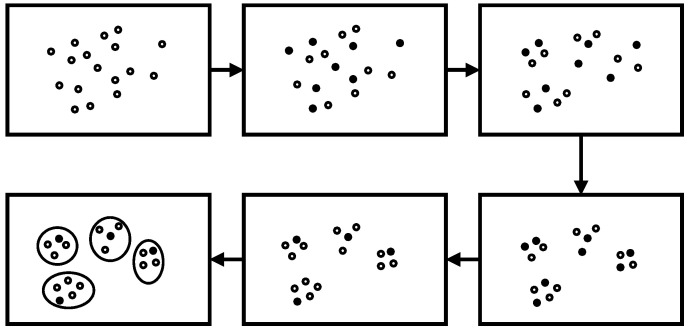
Estimation on the number of communities according to the evolution of community centers.

As shown in Figure 5, the solid points denote the initial selected candidate centers based on the density value. In general, the number of initial centers was much larger than the number of real communities. Under the framework of OCEA, with the iterative update of centers and attribute weights using Equations (8) and (11), respectively, the *l* step random walk probability changes and influences the similarity between vertices. In general, the vertices in the network gradually gather to several clusters in a distinct trend because of the adjustment of weights.

During the update of memberships, there may be several clusters that select the same vertex as their centers—that is, there may exist a vertex whose memberships to several clusters are all the highest in the current cluster. Only the vertices with the highest memberships are preserved as centers. This implies that parts of the candidate centers will be eliminated. As shown in Figure 5, parts of the solid points are transformed into hollow points, which implies that the solid points are no longer regarded as centers. After the elimination of centers, the number of remaining centers can be observed as an approximation of the number of communities. Function 1 summaries the process of cluster number estimation.
**Function 1.** ClusterNumberEstimation.**Input:** Attribute Augmented Graph *G_A_*(*V*, *E*, *A*, *X*), random walk length *l*, stop probability *c*, number of epochs *T***Output:** Number of Communities *k*1:Initialize weights *ω*_0_ = *ω*_1_ = … = *ω_d_* = 0, *d_S_* = 02:**for** vertices *v_i_* *∈* *V* **do**3: Compute density *D*(*v_i_*) by Equation (12)4: *d_S_* = *d_S_* + *D*(*v_i_*)5:**end for**6:*d_T_* = *d_S_*/*n*7:Initialize centers *V_D_* ← {*v_i_|D(v_i_)* ≥ *d_T_*, *v_i_* *∈* *V*}8:*L_k_* = |*V_D_*|9:**for***i* = 1, …, *T* **do**10: **for** vertices *v_i_* *∈* *V* **do**11:  Update memberships *U_ij_* and *X_c_* by Equations (6) and (7)12: **end for**13: **for** *m* = 1, …, *d* **do**14:  Update weights of attributes *ω_m_* by Equation (11)15: **end for**16: Compute *R_A_* by Equation (5)17:**end for**18:**for***j =* 1, …, *L_k_* **do**19: *V_C_* ← argmax*_vi_*(*U_ij_*)20:**end for**21:*k* = |*V_C_*|22:**return***k*

### 3.4. OCEA and AOCEA

As introduced in the previous sections, the update of memberships and the adjustment of weights are the main processes of OCEA, as shown in Algorithm 1.

It initializes weights, the random walk similarity, and centers first (lines 1–3). Then, the memberships, centers, and weights of attributes are updated iteratively until the convergence of the objective function in a certain range (lines 4–17). Detected communities are obtained using memberships (lines 18–23).
**Algorithm 1** OCEA**Input:** Attribute Augmented Graph *G_A_*(*V*, *E*, *A*, *X*), random walk length *l*, stop probability *c*, convergence parameter *ε*, overlapping parameter *γ*, number of communities *k***Output:** Communities *C*_1_, …, *C_k_*1:Initialize weights *ω*_0_ *= ω*_1_ = … = *ω_d_* = 12:Compute initial *R_A_* by Equation (5)3:Select the initial centers *V_C_*4:**while***mar* > *ε* **do**5: **for** vertices *v_i_* *∈* *V* **do**6:  **for** *j =* 1, …, *k* **do**7:   Update memberships *U*_ij_ by Equation (6)8:   Update centers *X_c_* by Equation (7)9:  **end for**10: **end for**11: **for** *m =* 1, …, *d* **do**12:  Update weights of attributes *ω_m_* by Equation (11)13: **end for**14: Compute *R_A_* by Equation (5)15: Compute objective function *F^t^*16: *mar* = |*F^t^ − F^t−1^*|17: *F^t−1^ = F^t^*18:**end while**19:**for** vertices *v_i_* *∈* *V* **do**20: *μ^i^_max_ =* max({*U_ij_*|*j* = 1, …, *k*})21: **for** *j =* 1, …, *k* **do**22:  *C_j_* ← {*v_i_|U_ij_ ≥ γ × μ^i^_max_*}23: **end for**24:**end for**25:**return** *C*_1_, …, *C_k_*

As shown in Algorithm 2. AOCEA first utilizes the function *ClusterNumberEstimation* to obtain an estimated number of communities *k*. Then, this estimated *k* is regarded as the input of the OCEA to detect the overlapping communities.
**Algorithm 2** AOCEA**Input:** Attribute Augmented Graph *G_A_*(*V*, *E*, *A*, *F*), random walk length *l*, stop probability *c*, convergence parameter *ε*, overlapping parameter *γ***Output:** Community *C*_1_, …, *C_k_*1:Initialize *k* = ClusterNumberEstimation (*G_A_*, *l*, *c*, *T*)2:return OCEA (*G_A_*, *l*, *c*, *ε*, *γ*, *k*)

The time complexity of computing the transition probability is ${O(|E}_{A}\left|\right)$. Here,$\;E_{A}$ denotes the set of edges in an attribute augmented graph that consists of two parts. The edges in an attribute augmented graph composed of two parts. One includes the original edges in the topological graph, the other includes the added edges according to the attribute information. Let $z_{p}$ be the numbers of nonzero entries in transition probability matrix *P* and *n* be the number of vertices. Since *P* is a sparse matrix, the time complexity of the matrix multiplication is ${O(z}_{p}^{2}n)$. Weight adjustment requires *O*(*nk*) time. Here, *k* is the true number of communities. Thus, the overall complexity of OCEA and AOCEA is ${O(|E}_{A}{|+z}_{p}^{2}n+nk)$, which can be reduced to *O*(*n*) because *k* << *n*. AOCEA has an extra procedure of estimating the number of communities, and its initial estimation of the number of communities is roughly equal to *n*/2. Therefore, the time complexity of weights adjustment in AOCEA is $O(n^{2})$ and gradually reduces to ${O(nk}_{e})$, where $k_{e}$ is the final estimation of the number of communities.

## 4. Experiments

### 4.1. Datasets

The algorithms were tested on synthetic and real-world attributed networks.

We evaluated the performance of proposed algorithms and the baselines on six real-world attributed networks. The Facebook egonetwork set (http://snap.stanford.edu/data/) (accessed on 28 April 2021) is a set of ten Facebook users’ ego networks. The users are denoted by nodeId’s and each ego network is numbered by the specific nodeId. Vertices denote the users of Facebook and each two pair of vertices within a circle (friends lists) are connected by an edge with a certain probability. The user profiles are encoded into vectors with binary values [40].

Texas, Washington and Wisconsin are three citation networks, where the vertices denote papers and the edges denote the citation relationships among the papers. The features of the papers are encoded into binary values. The papers are classified according to their domains [41].

Synthetic networks and the true communities were generated by the LFR benchmark [42]. The methods in [43] were used to generate each network’s attribute matrix according to its true communities. Specifically, the vertices in a community shared the same attributes with high probability while the probability for the vertices in different communities is low. The generated attributes were consistent with the format of .feat files of the real-world network Facebook.

Table 2 presents the meaning of the parameters for the LFR benchmark networks. Two sets of synthetic networks D_1_ and D_2_ were generated to evaluate the algorithms. D_1_ is a set of seven networks with increasing community size from 100 to 700. D_2_ is a set of five networks with increasing mixing parameters *μ*. The number of attributes was set to be the same as the number of communities, and *τ*_1_ and *τ*_2_ were set as default values 2.0 and 1.0, respectively. Table 3 lists the corresponding parameters of D_1_ and D_2_.

In addition to synthetic networks, three egonetworks from Facebook egonetwork set were also used in our experiments. As shown in Table 4, *N* is the number of vertices, *M* is the number of attributes, and *k* denotes the number of real communities.

### 4.2. Evaluation Metric

Overlapping normalized mutual information (ONMI) [44] is an improved measure of normalized mutual information (NMI) that can be used as the evaluation metric for overlapping community detection. This metric reflects the accuracy of the detected communities. The higher the value of ONMI, the higher the similarity between detected communities and ground-truth communities. Unlike NMI, ONMI can measure the accuracy of overlapping communities.

Given two covers, detected communities *X* and ground truth *Y*, H^*^(*X_i_*|*Y_j_*) is defined as

(13) $$H^{*}\left(X_{i}|Y_{j}\right)=\{\begin{array}{l} H\left(X_{i}|Y_{j}\right),\;\;\mathrm{if}\;h(a,n)+h(d,n)\geq h(b,n)+h(c,n)\\ h(c+d,n)+h(a+b,n),\;\;\mathrm{otherwise}\; \end{array}$$

where *a*$=\overset{n}{\underset{m=1}{\sum }}\left[X_{im}=0\wedge Y_{jm}=0\right]$, *b* = $\overset{n}{\underset{m=1}{\sum }}\left[X_{im}=0\wedge Y_{jm}=1\right]$, *c* = $\overset{n}{\underset{m=1}{\sum }}\left[X_{im}=1\wedge Y_{jm}=0\right]$, *d* = $\overset{n}{\underset{m=1}{\sum }}\left[X_{im}=1\wedge Y_{jm}=1\right]$ and $h\left(w,n\right)=log_{2}\left(w/n\right)$.

In Equation (13), *i* and *j* denote the index of the clusters. *X_im_* and *Y_jm_* denote whether there is a vertex *m* in the cluster *i* or *j*, respectively. Subsequently, the entropy *H*(*X*|*Y*) and *H*(*X*) can be obtained as

(14) $$H(X|Y)=\sum_{i\in \left\{1,\ldots K_{X}\right\}}\min_{j\in \left\{1,\ldots K_{Y}\right\}}H^{*}\left(X_{i}|Y_{j}\right)$$

(15) $$H(X)=\sum_{i=1}^{K_{X}}\left(h\left(\sum_{m=1}^{n}\left[X_{im}=1\right],n\right)+h\left(\sum_{m=1}^{n}\left[X_{im}=0\right],n\right)\right)$$

Next, *H*(*Y*|*X*) and *H*(*Y*) can be obtained similarly. ONMI is defined as

(16) $$\mathrm{ONMl}=\frac{\frac{1}{2}[H(X)-H(X|Y)+H(Y)-H(Y|X)]}{\max(H(X),H(Y))}$$

### 4.3. Experimental Scheme

Four experiments were conducted: runtime, the accuracy of community detection on synthetic or real-world networks, and the accuracy of the estimation of the number of communities.

The baseline methods include Inc-cluster, S-cluster, W-cluster [14], OCEA, and AOCEA. Inc-cluster utilizes random walk similarity as the similarity between each pair of vertices and k-means [45] to detect communities without considering overlapping communities. Its adjustment of weights does not consider the memberships of each vertex to communities. 

S-cluster only considers a network’s topology information, setting the structural weight *ω*_0_ to 1.0 and other weights to 0.0. W-cluster is an algorithm that considers attribute information without weight adjustment. It sets the structural weight to 1.0 and the weight of each attribute to 1/*d*. S-cluster and W-cluster can be regarded as the variants of Inc-cluster without adjustment of weights. The step length *l* is set to 5 for all algorithms. The power *m* in Equations (7) and (9) is set to 2. The *ε* is set to $10^{-5}$. The stop probability *c* is varying from 0 to 1:

1. Accuracy of detected communities on synthetic networks: Experiments were conducted on D_1_ and D_2_ to compare the accuracy of detected communities. D_1_ fixes the mixing parameter *μ* to 0.1 and sets the size of networks from 100 to 700. Meanwhile, D_2_ fixes the size of the networks to 600 and sets the mixing parameters *μ* from 0.2 to 0.6.
2. Accuracy of detected communities on real-world networks: Experiments were conducted on three networks from Facebook egonetwork set and three paper citation networks: Texas, Washington, and Wisconsin. The accuracy of the detected communities was studied and analyzed.
3. Accuracy of estimation on the number of communities: Experiments on Facebook egonetwork set, including all the ten egonetworks, to study the accuracy of the estimation on real-world networks.
4. Runtime: Experiments were conducted on D_1_ to compare the algorithm runtimes. As the size of networks increasing, we analyze the changing runtime trends.

The above experiments were conducted on a computer with an Intel i5-6300HQ CPU @ 2.30 GHz, with 8 GB RAM.

### 4.4. Results and Analysis

#### 4.4.1. Accuracy on Synthetic Networks

**Figure 6:**
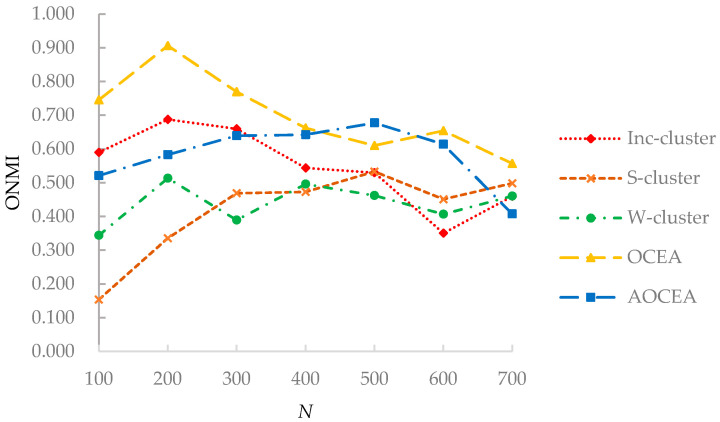
ONMI values of algorithms with varying network sizes.

**Figure 7:**
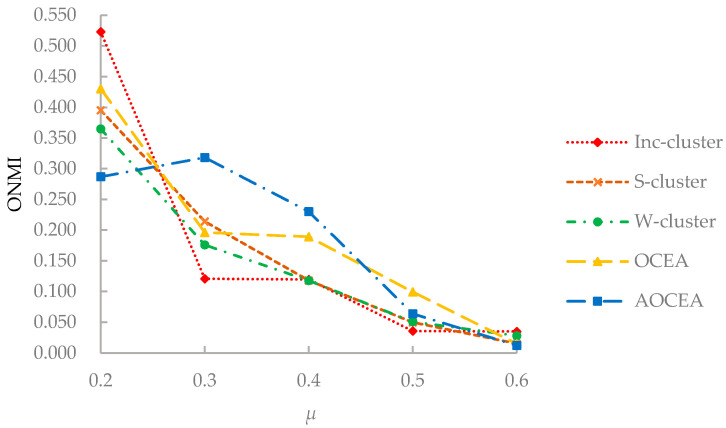
ONMI values of algorithms with varying values of *μ*.

The results of ONMI values on synthetic datasets D_1_ and D_2_ are shown in Figure 6 and Figure 7.

Figure 6 shows that the ONMI value of algorithms is maintained within a certain range with an increasing number of vertices. OCEA had a higher ONMI value on D_1_ than the other algorithms because they consider the vertex’s memberships. S-cluster detects communities without considering attribute information, and W-cluster does not have the process of attribute weight adjustment. Consequently, the superiority of OCEA and AOCEA reflects the effectiveness of considering attribute information and corresponding adjustment of weights. Besides, the adjustment of weights in OCEA and AOCEA considers the memberships of each vertex to communities. That is, the attribute weights of the vertices in communities are updated according to the memberships. Inc-cluster does not consider the attribute weights to communities in the adjustment of weights.

As shown in Figure 7, with the increase of the values of the mixing parameter *μ*, it becomes difficult for the algorithms to uncover the communities with increasingly blurred boundaries, which results in the decrease of the ONMI values. All algorithms could not detect communities correctly when the value of *μ* reaches 0.6. Although Inc-cluster [21] exhibited a high ONMI value at the beginning, it was greatly affected by the increase of the value of *μ*. S-cluster and W-cluster were affected less than other algorithms because of their inadequate use of attribute information. OCEA had a similar changing trend of the ONMI value compared to Inc-cluster because of the introduction of attribute information. The ONMI value of AOCEA decreased greatly when *μ* > 0.3 because it included an extra procedure of estimating the number of communities. The ONMI value of AOCEA increased when *μ* < 0.3 because the estimated number of communities is affected by the update of memberships matrix *U*. And the boundaries between communities are not blurred when *μ* < 0.3. The number of communities estimated by AOCEA may not be identical to the true ones when the value of *μ* is higher than 0.5, which affects its accuracy.

#### 4.4.2. Accuracy on Real-World Networks

**Figure 8:**
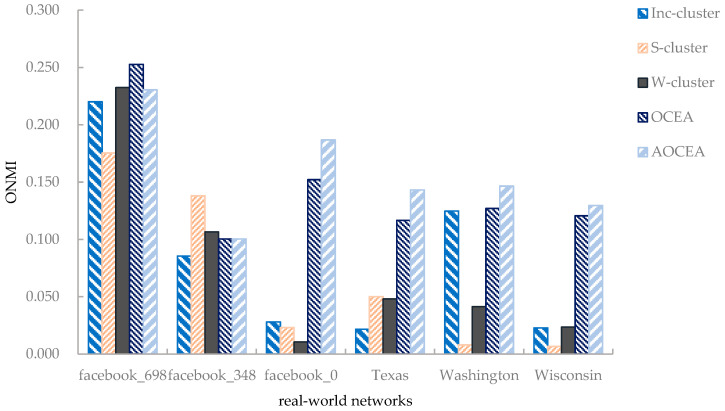
ONMI values of algorithms on real-world networks.

Six networks were selected to study the accuracy of algorithms on real-world networks. The results are shown in Figure 8.

As shown in Figure 8, the accuracy of OCEA was relatively higher than that of Inc-cluster algorithm [21] on the Facebook egonetworks. Although S-cluster only considers topology information, it still performs well on some networks. It is assumed that there may have been some attribute information that was not conducive to detecting communities—that is, the introduction of some attributes may have degraded the results of community detection. Moreover, the invalid attribute information may have influenced the estimation of the number of communities, resulting in the inferior performance of AOCEA on some networks. OCEA and AOCEA were superior to all the baselines because they consider the vertices’ community memberships and employ a strategy to adjust attribute weights iteratively according to the memberships. The vertices in the communities in the three paper citation networks (Texas, Washington and Wisconsin) are loosely connected, which results in the poor performance of S-cluster and W-cluster. Inc-cluster performed better than S-cluster and W-cluster because it considers the adjustment of attribute weights. However, it is not competitive with OCEA and AOCEA because it does not consider the vertices’ community memberships.

On the whole, as we can observe from Figure 6, network size has little impact on the algorithms’ accuracy when it is greater than 400. We conjecture that the accuracy of an algorithm on a synthetic network should exhibit little change when all network parameters in Table 2 except *N* are kept invariant because the more important structure and attribute features of the network are invariant. Additionally, as shown in Figure 8, the accuracy of an algorithm on a large network (facebook_0 with 347 vertices) may be inferior to that on a small network (facebook_698 with 65 vertices), which also reveals that other features of a network than its size may have a greater impact on its accuracy.

#### 4.4.3. Accuracy of Estimation on the Number of Communities

Experiments were conducted on all ten egonetworks from Facebook egonetwork set, and the number of vertices on each network was 347, 1045, 792, 755, 547, 227, 59, 159, 170, and 66, respectively. The results of the estimation of the number of communities are shown in Table 5. Here, real, initial, and estimation denote the real number of communities, the number of selected initial candidate centers, and the estimated number of communities by AOCEA, respectively.

As presented in Table 5, the number of initial candidate centers was much larger than the real ones. After the estimation, most of the estimated number of communities were generally close to the real ones except for facebook_1912, facebook_3437, and facebook_414. The gaps between the estimated and real number of communities on some networks were assumed to be caused by the sparse structure of communities on these networks.

#### 4.4.4. Runtime

**Figure 9:**
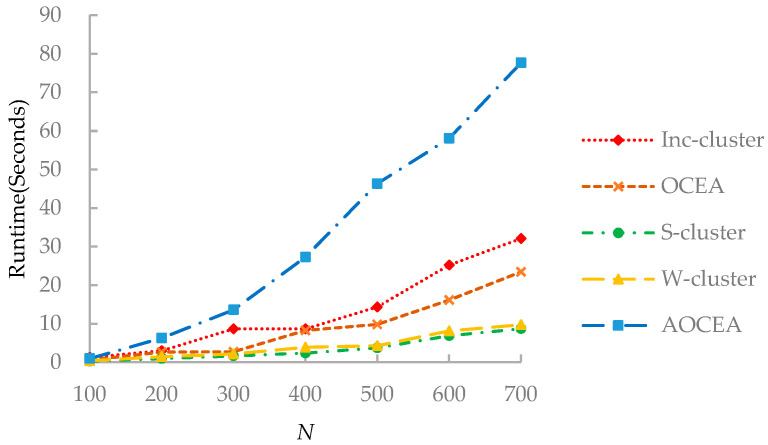
Runtime of the algorithms.

As shown in Figure 9, each algorithm exhibited a different trend with the increasing number of vertices. OCEA ran faster than Inc-cluster because the adjustment of attribute weights and the update of the centers in OCEA are more efficient than that in Inc-cluster. S-cluster and W-cluster methods do not contain the process of updating weights, leading to their fast running speed. The runtime of AOCEA is much higher than that of other algorithms because it contains an additional process to estimate the number of communities.

## 5. Conclusions

In this paper, an overlapping community detection algorithm called OCEA—based on an augmented attribute graph—and its extended version with an additional estimation process for the number of communities was proposed. To utilize the attribute information properly, we adopt a strategy of iteratively adjusting attribute weights. For overlapping community detection, fuzzy k-medoids [39] was employed. In addition, a process to estimate the number of communities was introduced to solve the community detection problems despite the number of communities being unknown. Experimental results showed the effectiveness of the proposed algorithms in real-world and synthetic networks. In the future, we will continue to study the strategy to estimate the number of communities more precisely to increase the performance of AOCEA. We will also explore new heuristic methods to conduct biased random walk towards vertices with high attribute weights to compensate for the inaccuracy caused by the loss of structural information in community detection on sparse networks. Furthermore, we plan to adopt approximate random walk to improve our algorithms’ performance on large networks.

## Figures and Tables

**Table 1:** Statistics of the vertex density values of the Facebook’s egonetworks.

Network	Mean	Variance	Standard Deviation
Facebook_686	${9.134\;\times \;10}^{-5}$	${2.294\;\times \;10}^{-9}$	${4.804\;\times \;10}^{-5}$
Facebook_414	${4.849\;\times \;10}^{-5}$	${6.579\;\times \;10}^{-10}$	${2.573\;\times \;10}^{-5}$
Facebook_698	${1.031\;\times \;10}^{-4}$	${3.079\;\times \;10}^{-9}$	${5.591\;\times \;10}^{-5}$
Facebook_3437	${7.135\;\times \;10}^{-6}$	${2.517\;\times \;10}^{-11}$	${5.021\;\times \;10}^{-6}$

**Table 2:** The meaning of parameters in LFR benchmark networks.

Parameter	Meaning
*N*	number of vertices
*k_avg_*	average degree
*k_max_*	maximum degree
*μ*	mixing parameters
*τ*_1_	negative exponent of degree
*τ*_2_	negative exponent of community size
*c_min_*	minimum community size
*c_max_*	maximum community size
*on*	number of overlapping vertices
*om*	maximum of an overlapping vertex belongs to

**Table 3:** Parameter settings of D_1_ and D_2_ datasets.

Dataset	Parameters
D_1_	*N* = 100–700, *k_avg_* = 10, *k_max_*=25, *μ* = 0.1, *c_min_* = 10, *c_max_* = 50, *on* = 20, *om* = 2
D_2_	*N* = 600, *k_avg_* = 10, *k_max_* = 25, *μ* = 0.2–0.6, *c_min_* = 10, *c_max_* = 50, *on* = 20, *om* = 2

**Table 4:** Information of real-world networks.

Network	*N*	*M*	*k*
Facebook_698	65	48	13
Facebook_348	225	161	14
Facebook_0	347	2533	24
Texas	187	328	5
Washington	230	446	5
Wisconsin	265	530	5

**Table 5:** Estimation on the number of communities in the Facebook’s egonetworks.

Facebook	Real	Initial	Estimation
facebook_0	24	127	17
facebook_107	9	417	17
facebook_1684	17	335	14
facebook_1912	46	338	12
facebook_3437	32	230	15
facebook_348	14	109	14
facebook_3980	17	27	14
facebook_414	7	85	19
facebook_686	14	76	8
facebook_698	13	28	16

## Data Availability

The data presented in this study are openly available in http://snap.stanford.edu/data/, accessed on 28 April 2021.
